# Extracellular Vesicles for Cancer Therapy: Impact of Host Immune Response

**DOI:** 10.3390/cells9010224

**Published:** 2020-01-16

**Authors:** Katie E. Gilligan, Róisín M. Dwyer

**Affiliations:** 1 Discipline of Surgery, Lambe Institute for Translational Research, National University of Ireland Galway (NUIG), H91 YR71 Galway, Ireland; k.gilligan3@nuigalway.ie; 2 CÚRAM, SFI Research Centre for Medical Devices, National University of Ireland Galway (NUIG), H91 W2TY Galway, Ireland

**Keywords:** extracellular vesicles, immune response, cancer

## Abstract

In recent times, extracellular vesicles (EVs) have come under the spotlight as potential therapeutics for cancer, due to the relative ease of manipulation of contents and potential for tumor targeting. The use of EVs as delivery vehicles may bypass some of the negative effects associated with cell-based carriers, and there has been a major focus on defining EV subtypes, establishing transparent nomenclature, and isolation and characterization techniques. EVs are believed to be a fingerprint of the secreting cell and so researchers harness the positive aspects of a particular cell of origin, and can then further modify EV contents to improve therapeutic efficacy. In this review, we highlight studies employing EVs as cancer therapeutics that have reported on immune response. As we rapidly advance towards potential application in the clinical setting, the question of immune response to EV administration in the cancer setting has become critically important.

## 1. Introduction

While there have been major advances in the field, the potential for activation of a toxic host immune response remains one of the major barriers to cell and gene therapy for cancer [1,2]. In recent years, there has been increased focus on the potential for extracellular vesicles (EVs) as gene delivery agents. EVs are secreted by all cells and while originally thought of a means of waste removal [3], are now known to play an import role in cell to cell communication, transporting genetic material between cells and into the circulation [3,4]. As a result, there is immense interest in EVs in the therapeutic setting for many diseases, including cancer [5]. Due to the relative early stage and rapid developments in the field, a major focus of EV research has been on refinement of isolation and characterization, nomenclature and classification of different EV subsets [6,7,8]. EV is a broad term encompassing several different subsets including exosomes, ectosomes, microvesicles and apoptotic bodies. Isolation and characterization of a single pure subset is difficult due to crossover in size and characteristics. Therefore, there have been attempts in recent years to set guidelines for researchers to allow for consistent, reliable and reproducible reporting of results, to support transparency and rapid advances in the field [6,7,8].

Immune response to EVs has been investigated extensively in relation to diseases of the immune system [9], however when administered in models of cancer most studies employ immunocompromised animals and do not investigate whether EVs initiate an immune response. It is most likely that EV immunogenicity depends on the model being used and the EV source and composition [10,11,12]. Given that EVs are thought to be a fingerprint of the cell source, if the cell induces an immune reaction when administered systemically, EVs may induce similar effects [13]. Thus, the EV cell source is a major consideration in studies, with researchers aiming to take advantage of the parental cell properties.

The effect of human EV administration in immunocompetent healthy animals was investigated by Zhu et al. 2017 [10]. EVs derived from wild type (WT) or engineered (miR-199a-3p) HEK293T cells were administered either intravenously (IV) or intraperitoneally (IP) three times weekly for 22 days and animals were sacrificed on day 23. Spleen cell immune phenotyping was performed targeting CD11b, CD11c, CD19, CD3^+^, CD4^+^, CD8^+^ with no significant changes observed. A few cytokines were altered, although not significantly and this was seen only in the engineered EV group, suggesting that content—miR-199a-3p—may have been detected by the immune system [10]. Overall this study shows the safe administration of human EVs and engineered EVs in immunocompetent healthy animals. In another study, crosstalk between immune cells, cancer cells and secreted EVs was investigated in oral tongue squamous cell cancer (OTSCC) cell lines HSC-3 and SCC-25 [14]. Initially, the effect of EVs isolated from the OTSCC cell lines on the cytotoxicity of CD8^+^ T and Natural Killer (NK) cells from healthy patients was investigated. It was found that the EVs increased cytotoxic activity of the cells, however the results varied depending on patient donor, EV parent cell and cancer cell type. In a zebrafish non tumor bearing model, EVs derived from these cell lines were administered, with decreased levels of anti-inflammatory Interleukin (IL)-13 reported. This was interesting as it was previously reported that IL-13 was increased in the saliva of OTSCC patients, suggesting that EVs derived from these cells do not cause this increase in IL-13 [14].

Mesenchymal stem cells (MSCs) have been extensively investigated for their immunosuppressive properties, and have been used in 67 clinical trials of inflammation associated diseases, transplant rejection and autoimmune diseases [15]. Originally it was thought that MSC therapy was dependent upon cell to cell contact, however it was discovered that suppression of T-cell proliferation could be accomplished by MSC-secreted factors alone [16]. Therefore, MSC-EVs are now being investigated to see if they hold the same properties as their parent cell [17]. MSC-EV immunomodulatory properties have been investigated, showing that EVs suppressed the secretion of pro-inflammatory Tumor Necrosis Factor alpha (TNF-α) and IL-1β in peripheral blood mononuclear cells (PBMC) but increased Transforming growth factor (TGF)-β concentrations in vitro [17]. Levels of regulatory T-cells and cytotoxic T lymphocyte-associated protein 4 were also increased. Interestingly, it was previously shown that indoleamine 2, 3-dioxygenase (IDO) mediated the immunoregulation by MSCs and an increase in IDO was seen in cells co-cultured with MSCs. However, no increase was seen when cells were co-cultured with the MSC-EVs. The study concluded that EVs and MSCs may differ in their immune-modulating mechanisms and activities [17].

## 2. Employing EVs to Promote an Anti-Tumor Immune Response

**Figure 1:**
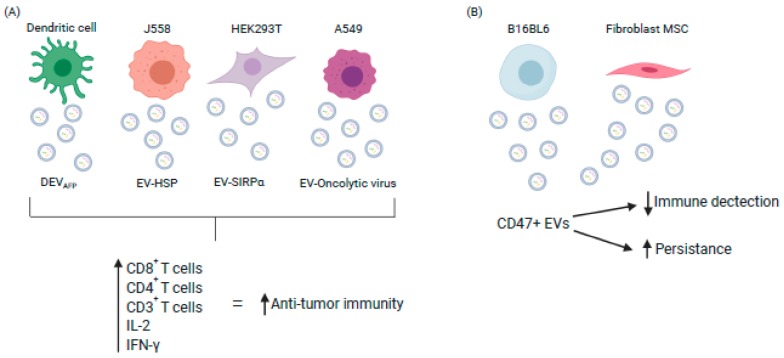
Exploiting EV characteristics to enhance or evade immune response (**A**) Engineered EVs stimulate immune cell infiltration into tumors and disease regression (**B**) CD47+ve EVs evade host immunity resulting in increased persistence and improved therapeutic response. (DEV-AFP: Dendritic EV α-fetaprotein; HSP: Heat Shock Protein; IL: Interleukin; IFN: Interferon. (Image created using Biorender.com—paid subscription).

Dendritic cell (DC)-derived EVs and their ability to trigger the immune response as a form of cancer therapy has been widely investigated. Three separate clinical trials have employed EVs as anti-cancer vaccines for the treatment of colorectal, melanoma or lung cancer. These studies showed that EVs were safe, non-toxic and tolerable, along with induced anti-tumor responses including induction of Cytotoxic T lymphocyte response and increased T cell activation [18,19,20]. Recently, Lu et al. [21] reported DC potential to treat hepatocellular carcinoma (HCC). EVs secreted by murine DCs expressing α-fetoprotein (DEV_AFP_), elicited a strong immune response when injected intravenously, which resulted in decreased tumor growth and increased survival rates for mice. There were significantly more CD8^+^ T lymphocytes, increased IL-2 and Interferon-γ (IFN-γ), with decreased TGF-β and IL-10. In nude and T-cell depleted animal models, there was lack of efficacy, suggesting that the T-cells contributed to the DEV_AFP_ mediated anti-tumor effect [21] (Figure 1). This study shows the potential positive effects of EVs triggering an immune response to treat HCC.

Studies have investigated the potential of using tumor-derived EVs (TEVs) to induce an anti-tumor immune response [22,23]. Myeloma cells were engineered to overexpress heat shock protein (HSP) 70 and the secreted EVs isolated (EV-HSP). Balb/c mice were immunized with the EVs to assess the P1A (tumor antigen)-specific T-cell response. EV-HSP simulated a type 1 CD4^+^ helper T-cell response. A P1A-specific CD8^+^ cytotoxic T lymphocyte response was also stimulated resulting in anti-tumor immunity [24].

Administration of TEVs as a vaccine has also been considered in a lung cancer model. EVs were isolated from a non-small cell lung cancer cell line (A549) overexpressing Rab27a. Mice were pre-immunized with EV-Rab27a for 2 weeks before being challenged with A549 cells, followed by EV-Rab27a for a further 2 weeks after administration of the cells. Tumor growth was significantly inhibited in the group that received EV-Rab27a compared to the controls. When the EVs were administered into a pre-established tumor model, tumor growth was also found to be inhibited [25].

The immune balance is regulated by immune checkpoint inhibitor molecules, and neutralization of these immunosuppressive checkpoints can lead to the elimination of cancer. Programmed Death-Ligand 1 is a membrane bound ligand found on the surface of many cell types and is up-regulated in tumor cells [26]. This cell surface expression is thought of as a means of immune evasion. PD-L1 binds to PD-1 on T-cells through its extracellular domain, suppressing activation of T-cells [27,28]. Inhibition of PD-L1 will allow more efficient T-cell response to cancer and based on this several therapies have now been approved [26]. Cancer cells have been found to secrete EVs with PD-L1 expressed on their surface. EV-PD-L1 has also been found to suppress T-cell function both in vitro and in vivo [26,28]. To knockdown EV-PD-L1 production, Poggio et al. [28] knocked out RAB27a, which resulted in the reduction of EV secretion and therefore a loss of EV-PD-L1. Murine prostate cancer cells TRAMP-C2WT, RAB27a knockdown (RAB27anull) or PD-L1 knockdown (PD-L1null) were injected into the flank of mice and monitored for 4 months. Mice that received either of the null cell populations showed no tumor growth and extended life span when compared to those administered with WT cells. When the immune response was investigated, CD8^+^ cells made up a greater fraction of the T-cells in the draining lymph node following injection of the two EV null cells compared to the WT, indicating an immune response initiated by the EVs. Mice receiving null cells had decreasing CD8^+^ and CD4^+^ cells that were PD-1 high. Interestingly, mice who had previously received the null cells were re-challenged 90 days later with WT cells and showed no tumor growth compared to the control group which had not been previously challenged. This demonstrated a robust memory response even against the WT cells that expressed EV-PD-L1. The study shows the ability of EVs to migrate to the draining lymph nodes and inhibit T-cell activation, and potential for EV-PD-L1 knockdown therapeutics [28].

Another study investigated EV-PD-L1 knockdown in melanoma [27]. Interferon (IFN)-α stimulation of A375 melanoma cells increased the amount of PD-L1 on the surface of secreted EVs. These EVs when injected IV then suppressed CD8^+^ T-cell function to facilitate tumor growth in a murine model of melanoma. The study showed that EV-PD-L1 bound to T-cells; moreover, EVs derived from IFN-α treated cells exhibited higher binding to CD8+ T-cells. In vivo B16-F10 cells with knocked down PD-L1 expression were subcutaneously injected into immune competent mice. EVs derived from B16-F10 WT cells were administered IV, which promoted tumor growth in the PD-L1 knockdown group. Pre-treatment with EVs incubated with anti-PD-L1 antibodies inhibited the effect. The EVs derived from B16-F10 WT were shown to decrease the proportion of proliferating PD-1-CD8^+^ T-cells in the spleen and lymph nodes, suggesting that EV-PD-L1 suppresses systemic anti-tumor immunity. Levels of PD-L1 on circulating vesicles in melanoma patients during anti-PD-L1 therapy were also examined, revealing that the pre-treatment level of circulating EV-PD-L1 was significantly higher in patients who did not respond to anti-PD-L1 treatment [27].

Yang et al. [29] investigated whether EVs could transfer PD-L1 to other cells. EVs were isolated from MDA-MB-231 and 4T1 breast cancer cells over-expressing PD-L1. EV-PD-L1 could be transferred to the negative MCF-7 and BT549-PD-L1^ko^ breast cancer cells, and also to other cell types including human myeloid antigen presenting cells and macrophages. They also showed the EV-PD-L1 bound to PD-1 on T-cells to inhibit T-cell activation and function. When EV secretion was blocked by Rab27a knockdown, there was inhibited tumor growth, showing similar results to the previous study [27]. This transfer of PD-L1 to other cells within the tumor microenvironment could contribute to tumor immune evasion [29]. Together these studies show the importance of EV-PD-L1 in the cancer setting.

CD47 has been shown to bind to signal regulatory protein alpha (SIRPα) and together they initiate a “don’t eat me” signal. This signal has been investigated for its ability to inhibit phagocytosis of tumor cells [30]. EVs derived from HEK293T cells were engineered with SIRPα on their surface (EV-SIRPα), in order to target and block CD47 in a colorectal cancer model. In an immunocompromised model bearing HT29 tumors, intratumoral administration of EV-SIRPα resulted in no significant decrease in tumor burden when compared to the control group. When EV-SIRPα were administered IV into an immunocompetent CT26.CL25 mouse model there was a dramatic reduction in tumor growth [30]. This suggests that the immune system played a role in the therapeutic response. When analyzed further, it was discovered that there was extensive CD8^+^ T-cell infiltration when compared to the control animals. These data suggest that the engineered EVs could prevent CD47-mediated tumor evasion of an immune response and aid in therapeutic efficacy [30].

## 3. Employing EVs to Increase Persistence and Targeting of Therapeutics

While in a previously mentioned study, EV-SIRPα were employed to block the activity of tumor cell CD47 and promote an anti-tumor immune response, CD47 expression on EVs is also thought to be the mechanism by which EVs go undetected by the immune system. This is important regarding the therapeutic setting where EV rapid clearance would reduce efficacy. Balb/c mice received gLuc-LA- (Gaussia luciferase–lactadherin) labelled B16BL6 (murine melanoma) EVs at varying doses. Macrophage depleted mice were prepared by administrating clodronate liposomes, followed by labelled EVs administered IV. The rate of gLuc activity declined much slower in macrophage-depleted mice, with a serum EV concentration 285-fold higher than those of the untreated mice, indicating that macrophages play a key role in EV clearance. Ideally going forward, EVs from other cell sources would be investigated to see if macrophages process all EV types in this manner [31].

EV-CD47 has also been investigated in a model of pancreatic cancer, where mutated forms of KRAS are commonly seen [32]. EVs and liposomes derived from both human and murine normal fibroblast-like mesenchymal cell lines were loaded with Alexa Fluor 647 (AF647) tagged siRNA, targeting oncogenic KRAS. When IP injections were performed, the iEVs (AF647-siRNA loaded EVs) but not iLiposomes (AF647-siRNA loaded liposomes) were detected in the circulation of both nude and immunocompetent animals 24 h later. Interestingly, iLiposomes enhanced the mobilization of CD11b^+^ monocytes in the circulation, however this effect was not seen with the iEVs. The levels of CD47 on EVs had an inverse correlation with circulating AF647^+^ monocytes, further supporting that CD47 prevents EV clearance (Figure 1). In CD47 knockout mice, there were significantly less EVs in the circulation after administration. Animals treated with IP iEVs had significantly reduced tumors compared to the control groups, with the disease being barely detectable and animals surviving 200 days after treatment. When CD47 was blocked using an antibody on the EVs, it was found that there was no reduction of tumor burden when compared to the iEVs. Together, these data support that CD47 presence on EVs contributes to evasion from host immune clearance [32]. This study shows promise for EV use in the therapeutic setting.

Oncolytic virus therapy involves viruses that have been specifically engineered to infect, replicate and kill preferentially in cancer cells, while in normal cells the activity is restricted. Due to safety concerns, experimental oncolytic viruses are generally delivered in preclinical models via local administration. The disadvantage of virus administration is the potential for immune detection and inactivation thus preventing viral replication and spread in cancer cells. Garofalo et al. [33] first investigated the effects EV encapsulated oncolytic virus to prevent immune detection and allow for IV administration to treat lung cancer. EVs secreted by A549 cancer cells infected with the oncolytic virus (EV-Virus) were isolated and loaded with paclitaxel (EV-Virus-PTX). Immune compromised mice with subcutaneous A549 lung cancer were treated with EV-Virus-PTX, EV-Virus, or EVs alone. It was reported that EV-virus-PTX had enhanced therapeutic effect when administered IV with significant tumor reduction compared to IT.

This work on oncolytic viruses subsequently advanced to an immune competent model using murine EVs and tumors, again encapsulating the virus in EVs to protect it from immune recognition [34]. Murine EV-Virus (1 × 10^8^) labelled with a fluorescent dye (DiIC18) were administered either IP or IV. Subsequent imaging revealed a tumor-localized signal only in cases where the EVs were administered IV, but not IP. The study also investigated the tumor-associated immune response to the EVs. EV-Virus or inactivated free virus were administered IV or IP. IV administration of the EV-Virus resulted in infiltration of CD3^+^, CD4^+^ and CD8^+^ T-cells in the tumor similar to the virus alone group. The EVs did not influence the immune modulatory effects of the virus [34]. These studies together show the potential of EV encapsulated viruses as a targeted delivery system for oncolytic viruses.

Expression of Nucleolin is elevated on breast cancer cells and a DNA aptamer (AS1411—an oligonucleotide that binds to nucleolin) targets and binds to the phosphoprotein. AS1411 has shown inhibition of tumor activity and low systemic toxicity in a Phase II clinical trial [35]. EVs were isolated from murine dendritic cells, mixed with AS1411 overnight to coat the EVs, and then EV-AS1411 were engineered by electroporation to carry miR-let-7 [36]. Immunocompromised mice received IV EVs-AS1411-let-7 or conjugated AS1411-let-7. Mice that received EV-AS1411-let-7 demonstrated better therapeutic response. The group receiving EVs had significantly inhibited tumor growth when compared to the AS1411-let-7 or PBS groups, with enhanced tumor targeted delivery. Immune response to the engineered EVs was investigated. Serum associated cytokines IFN-α and TNF-α were measured in mice treated with EV-AS1411-let-7, with no statistically significant difference found when compared to a PBS treated group [36].

## 4. Discussion

Although not yet administered in cancer patients, MSC-EVs have been administered in a clinical trial for GvHD, after the failure of standard treatments [37]. Patients received seven escalating doses of EVs over two weeks, PBMCs were isolated and cytokine response monitored. TNF-α, IL-1β and IFN-α producing PBMCs were reduced by >50% following the final MSC-EV administration. During therapy, there was a reduction in pro-inflammatory cytokines IL-8, IL-6 and IL-17A. Clinical symptoms for the patients also improved including reduced vomiting and nausea which was stable 16 weeks post therapy [37,38]. MSC-EVs were also administered in a clinical trial setting for acute and chronic kidney disease. Twenty patients received two doses of MSC-EVs and another twenty patients received a placebo. Patients that received MSC-EVs had a significant improvement in kidney function, and increased plasma levels of IL-10 and TGF-β1 with decreased levels of TNF-α. Both studies demonstrate safe administration of MSC-EVs along with improvements in the target clinical parameters [37,39]. This has yet to be achieved in the cancer setting.

The generation and administration of clinical grade EVs for cancer treatment was recently reported in a pre-clinical study by Mendt et al. [40]. While previous studies have been limited in timeline, here the effect of long-term administration of EVs in an In Vivo model of pancreatic cancer was determined. EVs isolated from BJ fibroblasts (normal foreskin cells) were injected IP into immune competent mice every 48 h over the course of 4 months. Control mice received PBS, with no significant changes in immune response noted between the two groups. Another group of immune competent animals received either liposomes, BJ-EVs, MSC-EVs or iEVs (engineered as previously described by this group [32]) through IP injection every 48 h for 3 weeks. When immunotyping of the spleen, bone marrow and thymus was performed, no significant change in lymphocytes or myeloid cells was seen regardless of EV source. Circulating levels of IL-6 and IFN-α were not elevated in any of the groups, with levels remaining well below what is considered immunostimulatory. EVs were isolated by ultracentrifugation which is known to include co-isolated fractions, with no immune response observed, showing the isolation technique did not affect the study outcome [40]. These preclinical findings are promising for the use of EVs in the cancer therapeutic setting.

Many efforts have been made to increase EV secretion and yield including modifications in cell seeding density, cell immortalization, and the use of bioreactors applying mechanical shear stress [41]. Large scale production of EVs will be required for clinical application as current isolation methods produce low yields [40]. There are many other aspects of EV biology that need to be further understood to support application in clinical oncology, including factors controlling migratory itinerary and recipient cell uptake, and the impact of genetic modification. Although there has been a great expansion of EV research and ensuing publications in recent times, the studies highlighted here are the relatively few that provide any detailed insight into EV interaction with the host immune system. Together these studies show the importance of cell source in the therapeutic setting and the implications the secreted EV properties can have on the immune system. It is imperative that researchers address and report the potential EV-host immune interactions at an early stage in development of therapeutic EVs for translation to the clinical setting. This review emphasizes the need for careful selection of the parent cell for EV production, and the potential impact of engineering EV surface and cargo on immune response to the vesicles. The immune response will play a critical role in EV persistence in circulation and therapeutic efficacy.
